# An assessment of the IQAS discrete analyser for use in a routine water quality laboratory

**DOI:** 10.1155/S1463924684000079

**Published:** 1984

**Authors:** S. Wilson, A. Green

**Affiliations:** Finham Regional Laboratory Severn Trent Water Authority Coventry UK


Journal of Automatic Chemistry, Volume 6, Number 1 (January–March 1984), pages 33–38

### An assessment of the IQAS discrete analyser for use in a routine water quality laboratory

**S. Wilson and A. Green**
*Finham Regional Laboratory, Severn Trent Water Authority, Coventry, UK*

### Introduction

The instrument is a computer-controlled, high-speed, discrete colorimetric analyser capable of performing up to 20 different analyses sequentially with only minimal operator involvement.

### *Working units*

The sample wheel has 99 usable spaces for standards and samples in 2 ml autoanalyser cups, which are held in a thermostatically controlled water-bath at 8°C. The reaction wheel has 99 usable spaces for 6 ml glass reaction tubes, which can be thermostatically controlled between 30 and 50°C using the built-in water-bath. The sample arm is driven by a stepper motor and is pneumatically operated for transferring samples from the sample wheel to the reaction wheel. At the reaction wheel, sample is washed out of the pick-up line with diluent. Before resampling, the outside of the pick-up line is washed and dried in an intermediate position between the two wheels to minimize carry-over.

Sample sizes can be varied from 10 $\mu$l to 500 $\mu$l and larger samples can be taken by repeat sampling from the same cup.

Permanent storage is available for up to 60 reagents, in volumes varying from 100 ml to 1 l, 31 of which are refrigerated to 8°C. All the reagents are permanently piped, via electrically operated valves, to one of 27 dispense stations situated around and above the reaction wheel. Up to six reagents per test may be dispensed at any one of the 27 stations.

The reagent bottles are pressurized to 7·5 p.s.i. with compressed air and during a test the computer opens selected valves for fixed time intervals, allowing accurately measured volumes of reagents to be dispensed into the reaction tubes.

To ensure complete mixing of reagents and sample, two dual mixer stations are positioned around the reaction wheel. These are pneumatically driven air bubbling mixers and for each test the computer may be programmed with information on: (1) the use of one, two or no mixers; and (2) the size of the mixing bubbles produced and the time interval between bubble production.

At the photometer station coloured solution is transferred from the reaction tubes into the photometer for concentration measurement. For any test the computer will select: (*a*) the most suitable interference filter (20 available); (*b*) the volume of sample drawn through the photometer before the solution is held stationary and an absorption measurement is made; (*c*) acceptance limits for the number of separate measurements (usually three) made on each solution, and (*d*) whether the photometer station is used on the first or subsequent passes (in order to complete complex chemistries and to allow time for intermediate chemical reactions to occur, it is possible to permit the reaction wheel to rotate more than once before transfer to the photometer [the maximum number of revolutions is four]; similarly it is possible to arrange for mixing and reagent addition to occur on any or all of the passes).

### *Operation*

After test selection, which may be manual or automatic, the following sequence is followed for each analysis:

(1) The computer requests the number of samples to be analysed (this stage is automatic if automatic test selection [ATS] is used).
(2) The computer advises the number of reaction tubes required to complete the test (including wash tubes and five standards). These are then loaded by the operator.
(3) The operator presses 'check list' (final check on numbers of samples, standards and tubes required), then 'Test start'.
(4) Wash water and reagents alone are added to the first four tubes and standards and samples plus reagents are added to subsequent tubes. The last two tubes contain only water, added automatically at station one, so that the mixers, the transfer probe and photometer are washed at the end of the test. (The purpose of the four wash tubes is to ensure that the lines leading from reagent bottles via valves to the dispense stations have been thoroughly flushed before standards and samples are begun. This is particularly important with refrigerated reagents. However, if the volume dispensed is small then the four wash tubes may be insufficient to flush the lines fully. Using a particular operating mode it is possible [with certain restrictions] to ensure that larger volumes are dispensed into some, or all of the wash tubes than are dispensed into the standards and samples thus ensuring complete flushing of the lines.)
(5) At the photometer station the samples are transferred and then absorbance values at the chosen wavelength measured.
(6) The computer displays diagrammatically the calibration graph produced by the five standards and shows optical density values for their colour intensity. The results for each sample is displayed in concentration units calculated from the calibration graph using point-to-point linear interpolation.

### *Operating system*

The operating system allows up to eight pre-set checks to be entered automatically anywhere in an ATS run. The computer keeps a continually updated file for each check and displays 1, 2 and 3 SD and CV for each check result. Pre-set standard values and concentration limits for each chemistry can be set such that 'undersize', 'oversize' or 'off-range' results are

*Table 1. Performance characteristics.*

| Test | Range (mg/l) | Throughput (tests/h) | Time for 40 samples (min) | Carry-over (%) | Detection limit (3 $\sigma$ mg/l) | CV (%) within batch $0{\cdot}75_R$ | CV (%) within batch $0{\cdot}25_R$ | % recovery standard addition |
|---|---|---|---|---|---|---|---|---|
| Ammonia | 0–30 | 360 | 14·2 | 0·09 | 0·02 | 0·4 | 0·5 | 100 |
| Ammonia* | 0–1·0 | 360 | 14·2 | 0·01 | 0·002 | 0·25 | 1·1 | 100 |
| Total oxidized nitrogen | 0–30 | 600 | 18·5 | 0·2 | 0·07 | 0·4 | 0·7 | 100 |
| Total oxidized nitrogen* | 0–5·0 | 600 | 18·5 | 0·35 | 0·015 | 0·25 | 0·4 | 100 |
| Chloride | 0–300 | 600 | 8·5 | 0·16 | 0·16 | 0·18 | 0·2 | 100 |
| Phosphate | 0–20 | 240 | 21·25 | 0·72 | 0·026 | 0·4 | 1·2 | 99 |
| Phosphate* | 0–2·0 | 200 | 25·5 | 0·5 | 0·004 | 0·5 | 0·5 | 101 |
| Sulphate | 0–200 | 600 | 8·5 | 0·2 | 0·18 | 0·7 | 2·0 | |
| Calcium hardness | 0–600 | 600 | 8·5 | 0·22 | 3·0 | 0·7 | 0·6 | 98–100 |
| Magnesium hardness | 0–100 | 600 | 8·5 | 0·29 | 0·3 | 0·6 | 0·6 | |
| Total hardness | 0–400 | 600 | 8·5 | 0·4 | 1·5 | 0·4 | 0·6 | |
| Silicon | 0–30 | 600 | 18·5 | 0·14 | 0·7 | 0·7 | | 100 |
| Nitrite | 0–5·0 | 600 | 8·5 | 0·23 | 0·05 | 0·7 | 0·7 | |
| Nitrite* | 0–1·0 | 600 | 8·5 | 0·5 | 0·006 | 0·4 | 0·6 | |
| Total hardness* | 0–80 | 600 | 8·5 | | 1·3 | 0·6 | 1·1 | |

* = Low-level method.

automatically flagged. It allows for a pre-set test sequence so that labile determinands can be done first and the rest in any order which suits the operator. Current work-lists can be viewed or printed at any time.

Once a test has been run the results are stored in a file for acceptance. At this stage one or all results can be rejected and automatically rescheduled.

The results are stored on the results (D1) disk in report form and can be viewed and altered at any time.

### Method development

The instrument, as supplied, has a package of water chemistries stored on disk, these cover most of the determinands normally measured, and a number of blank files into which additional methods may be written.

Existing methods may be modified through the 'keyboard utility'. Using this computer utility such things as the sample and diluent volumes, the reagent volumes, positions of addition, and volumes transferred for colorimetric measurement may be altered. It should be noted, however, that once changed the new conditions are immediately over-written on the operating (DO) disk and the old conditions lost. Great care should therefore be exercised in the use of this utility and safeguards, such as spare copies of operating disks (DO) and hard copies of operating conditions for each method, are essential.

The writing of entirely new methods into spare files involves the use of 'mark-sense' cards and the card-reader unit which is supplied with the instrument. For each new method four mark-sense cards are required, these program the computer with all the parameters over which the operator has control. The first line of each of the four cards is a card identity number so that the card reader can recognize it, and the last line contains a check sum which ensures that all the marks on the card have been correctly read. The new file is written by entering the cards (face down) in any order; minor changes to the method are made with the keyboard utility.

### Analytical methods

A brief indication of each method is as follows:

Nitrate—reduction to nitrite by copper/hydrazine catalyst. Sulphanilamide/colour reagent [1].

Ammonia—alkaline phenate/hypochlorite reaction with nitroprusside as catalyst [2].

Chloride—mercuric thiocyanate/ferric nitrate method [3].

Phosphate—ammonium molybdate method [4].

Sulphate—barium sulphate/turbidimetric method [5].

Silica—ascorbic acid/ammonium molybdate [6].

Nitrite—sulphanilamide reagent [1].

Calcium—O-cresolphthalene [7].

Magnesium—calmagite/EGTA [5].

Total hardness—calmagite/magnesium disodium EDTA [8].

### Method assessment

This was divided into two categories: assessment of the performance of the methods developed; and comparison of the results obtained with those from standard procedures (usually existing dynamic flow methods). The performances of the methods developed is shown in table 1. The inclusion of a column for 'time for 40 samples', as well as 'throughput', is intended to show the real time for analysis. It includes all the standards, blanks, checks and washes that would be required for 40 samples and allows for time of travel of the reaction wheel from the sampling point to the photometer transfer station.

The 'carry-over' tests were performed using full-scale and 10% full-scale standards run in groups of three. Five batches of alternate high and low groups of standards were run for each test. If each group of high standards is numbered $a_1$, $a_2$ and $a_3$ and each group of low standards $b_1$, $b_2$ and $b_3$, then the carry-over is:

$$\frac{\bar{x}b_1 - \bar{x}b_3}{\bar{x}a_3 - \bar{x}b_3} \times 100.$$

The detection limits (3 $\sigma$) were calculated using the mean of not less than 20 blanks. Coefficients of variation were calculated at three-quarters and one-quarter of full scale. Percentage recoveries were calculated by selecting samples and adding two 'spikings' of standard solution, one of which was approximately equal to the amount of determinand already present and one of approximately five times the amount present. The 'selection' of samples was necessary to keep the five times spiking within the full-scale range of the method.

*Table 2. Harmonized monitoring tests.*

| Method | | Standard solution X | Standard solution Y | River | Spiked river |
|---|---|---|---|---|---|
| TON | Sw | 0·03435 | 0·04658 | 0·04243 | 0·05495 |
| (0–30) mg/l | Sb | 0·04336 | 0·06573 | | |
| Total oxidized | St | 0·05532 | 0·0805 | 0·06482 | 0·1355 |
| nitrogen | Conc. | 5·07 | 9·82 | 6·74 | 16·71 |
| Experimental | $St^2/Z^2$ | 0·05 | 0·03 | 0·04 | 0·03 |
| Calculated | f0·05 | 1·69 | 1·75 | 1·69 | 1·69 |
| Ammonia | Sw | 0·01483 | 0·02598 | 0·00806 | 0·05749 |
| (0–30) mg/l | Sb | 0·03496 | 0·14230 | | |
| | St | 0·03797 | 0·14465 | 0·02011 | 0·16471 |
| | Conc. | 3·62 | 11·99 | 1·62 | 13·58 |
| Experimental | $St^2/Z^2$ | 0·04 | 0·06 | 0·06 | 0·06 |
| Calculated | f0·05 | 1·83 | 1·83 | 1·83 | 1·83 |
| Chloride | Sw | 0·18 | 0·37 | 0·19 | 0·39 |
| (0–300) mg/l | Sb | 0·23 | 0·62 | | |
| | St | 0·23 | 0·62 | 0·28 | 0·54 |
| | Conc. | 22·99 | 169·2 | 62·5 | 112 |
| Experimental | $St^2/Z^2$ | 0·04 | 0·005 | 0·008 | 0·009 |
| Calculated | f0·05 | 1·6 | 1·72 | 1·67 | 1·67 |
| Soluble phosphate | Sw | 0·066 | 0·063 | 0·036 | 0·063 |
| (0–20) mg/l | Sb | 0·082 | 0·114 | 0·066 | 0·100 |
| | St | 0·074 | 0·092 | 0·050 | 0·102 |
| | Conc. | 4·2 | 16·8 | 7·3 | 16·77 |
| Experimental | $St^2/Z^2$ | 0·124 | 0·012 | 0·02 | 0·015 |
| Calculated | f0·05 | 1·61 | 1·69 | 1·68 | 1·72 |

Where $St^2/Z^2 = \dfrac{\text{total standard deviation squared}}{5\% \text{ of the concentration squared (or 0·025)}}$.

In addition to the above tests, the recommended harmonized monitoring procedure was carried out. This involves measuring a blank, two standard solutions at high and low levels, a river and spiked river in duplicate on 10 different occasions in random order. This gives a within-batch, between-batch and total standard deviation, which can be compared to expected values at the 0·05 probability level (see table 2).

A minimum of 100 results were compared with those obtained using current dynamic flow systems and a mean percentage difference was obtained, a *t*-test was carried out also (see table 3).

Finally, having run the machine operationally for three months, there are CVs now for the total error (both within- and between-batch) on the controls for most of the determinands (see table 4).

*Table 3. Comparison of methods.*

| Determinand | Mean percentage difference | *t*-value | Degrees of freedom | Significance at the 0·01 level |
|---|---|---|---|---|
| Chloride (0–300) mg/l | 1·09 | 3·8 | 84 | * |
| TON (0–30) mg/l | 0·58 | 3·3 | 96 | * |
| Ammonia (0–30) mg/l | − 1·3 | −0·55 | 70 | NS |
| Calcium (0–600) mg/l | 0·37 | 1·3 | 55 | NS |

*Table 4. Analytical quality control data.*

| Determinand | No. of results | Mean conc. (mg/l) | Actual conc. (mg/l) | 1 SD | Total error CV (%) |
|---|---|---|---|---|---|
| Chloride 0–300) mg/l | 52 | 240·7 | 240 | 1·74 | 0·71 |
| Ammonia (0–30) mg/l | 56 | 24·88 | 25 | 0·18 | 0·70 |
| TON (0–30) mg/l | 49 | 22·52 | 22·5 | 0·18 | 0·79 |
| TON (0–30) mg/l | 20 | 5·15 | 5 | 0·08 | 1·5 |
| Chloride (0–300) mg/l | 51 | 51·45 | 50 | 0·92 | 1·7 |
| Sulphate (0–200) mg/l | 26 | 175·7 | 175 | 2·76 | 1·5 |
| Phosphate (0–20) mg/l | 54 | 15·09 | 15 | 0·12 | 0·77 |
| Ammonia (0–1·0) mg/l | 21 | 0·796 | 0·8 | 0·014 | 1·7 |
| Phosphate (0–2·0) mg/l | 20 | 1·524 | 1·5 | 0·015 | 0·96 |

### Sample processing

Samples arrive at the laboratory and are assigned automatic test selection (ATS) numbers. They are 'booked' into the laboratory computer (a Wang MVP 2200 system) via a sample reception program which records the ATS number and the analysis suite and/or determinands requested. The Wang computer is capable of memory partition. The initial sample reception step is carried out in Partition 1, subsequently the computer goes onto Partition 2 and continues with the rest of the sample reception program, i.e. determinand files are updated, worksheets are produced for other analyses carried out in the laboratory, and sample result files are created.

In Partition 1 mode, information on those samples requiring discrete analysis is transmitted by cable to the IQAS. This consists of the sample number, name and determinands requested. The IQAS automatically proceeds to do the analysis requested in a predetermined order. Results are shown on the VDU and appropriate action can be immediately taken. Results, when accepted, are stored on the D1 disk on the IQAS.

On completion of the analysis, or at some appropriate point, the Wang requests the results and stores them in the previously opened sample result files.

Results outside predetermined limits are rescheduled by the Wang and the process is repeated.

When all the analyses on a sample are completed they are automatically presented for validation and a report is produced.

The system has several major advantages over the previous system.

(1) Results are seen on generation, so appropriate action can be taken immediately.
(2) Once the sample number and analysis requested are entered into the computer there are no further transformations carried out, thus minimizing possible errors—an analysis run is scheduled, transmitted to the IQAS and stored on the Wang disk. The IQAS automatically completes the scheduled analysis run. The Wang requests the results for that analysis run and stores them into the previously opened files. Any discrepancies would be immediately apparent and would be flagged by the computer. In the previous system there were several transformations from computer to worksheet, worksheet to magnetic tape, magnetic tape back to computer, and then computer back to paper.

### Discussion

### *Total oxidized nitrogen*

The method adapted for the IQAS is the standard one in which nitrate present is reduced to nitrite by alkaline hydrazine with a copper catalyst. The resultant total nitrite is used to diazotize sulphahilamide which is then coupled with NEDD. The intensity of the dye produced is measured colorimetrically.

The method as initially supplied was not satisfactory. When tested for precision the CV was large (1·1%) and the reaction mixture over-reduced as measured by nitrite standards. Increasing the concentration of alkali or colour reagent above certain minimum levels by small amounts had no effect on the overall reaction. Several factors were varied to compensate for the over-reduction, whilst also trying to improve the precision: the reduction time; the ratio of colour development to reduction time; reductant strength; sample size; and temperature.

The calibration curve was found to be an 'S' shape, indicating over-reduction at low concentration and under-reduction at high concentration. By reducing the reductant strength the 'S' shape could be minimized to the point where 100% reduction was occuring as compared with nitrite standards. Then by reducing the sample size and, again, following the above procedure, the 'S' shape could be further straightened. The reaction temperature was also reduced from 40°C to 37°C to lower the reduction rate.

At 20 $\mu$l sample size precision was lost. This was due to two factors: the low sample size and low reductant strength. To improve precision, all volumes were doubled. It was also found that double mixing after reductant addition improved precision. This was presumably due to the need to completely mix the low-strength reductant mixture as soon as possible after addition. (In single mixing alternate tubes have to wait two positions before being mixed.)

As the calibration curve was almost linear ($r = 0{\cdot}9996$), and because exhaustive tests showed it to be quite reproducible, it was felt that further modifications were unnecessary. The precision had also been improved such that the CV at 75% of full scale was 0·4%.

In conclusion, it appeared that the 'S' shaped calibration curve was caused by over-reduction at low concentrations and under-reduction at high concentration. This was confirmed by comparison with nitrite standards at both ends of the calibration curve.

It was found that performing the reduction in more dilute solution minimized this effect. Precision limitations caused by small sample sizes and lack of final colour development prevented complete linearity from being achieved.

Because of the number of factors which can affect the performance of this method in routine operation it was thought advisable to have high and low nitrate standards and a nitrite standard included as controls in each routine run.

### *Ammoniacal nitrogen*

The method as adapted for the IQAS incorporates the standard alkaline phenate method in which an indophenol, blue colour is formed. This method had to be developed using mark-sense cards, as the IQAS used the salicylate method which gave unacceptably high blanks using the reagents available.

The method was initially formulated as a two-pass chemistry to simulate the dynamic flow system, where the mixing and order of addition are important. EDTA was added at station 1 and the alkaline phenate at station 9, just prior to mixing. The hypochlorite and nitroprusside catalyst were added at station 1A and mixer 2 was used. Mixer 1 was again employed on the second pass as a final colour mix. This method gave low ammonia results compared to the autoanalysers and the precision was not as good as expected.

The amounts and strengths of the reagents added were varied and in the case of the phenate, hypochlotrite and nitroprusside, small changes above certain minimum levels were found to give no improvement in the method. The EDTA buffer, however, was found to be quite inadequate, and after much experimentation an alkaline phosphate buffer was found to give greater sensitivity, a much reduced blank value and improved recovery of added ammonia. This was attributable to a much faster reaction rate at the optimized pH.

Experiments with changing the dispense positions were then undertaken on the assumption that ammonia loss could be occurring due to air mixing, or, possibly, after addition of a particular reagent. It was found that immediate air mixing after hypochlorite addition did indeed cause ammonia loss. Leaving too long a time interval after hypochlorite addition before air mixing, however, also caused precision problems, presumably due to incomplete mixing.

Optimization was eventually achieved by adding the buffer at station 1 and all other reagents at station 2. Adding the reagents together gives good mixing and there is no ammonia loss due to immediate air mixing. The reaction also occurs immediately after sample addition which again minimizes ammonia loss. The index time was varied to find the minimum time in which the maximum stable colour developed (10 s). Under this regime, in which only mixer 1 is used, the chemistry becomes single pass, with subsequent time saving.

It was also discovered that there was ammonia loss from the samples on the sample wheel. Thus the test must be carried out as soon as practicable ($\frac{1}{2}$ to 1 h) after sampling and should be scheduled first in any multiparameter run.

In conclusion, unlike dynamic flow systems, the order of reagent addition is unimportant. Also, unlike autoanalysers, where the system is enclosed ammonia loss can be a problem. This has to be minimized by carrying out the reaction immediately after sample addition and avoiding air mixing until the labile ammonia is chemically bonded. The reaction is pH-dependent.

### *Other methods*

Calcium gives a colour with O-cresolphthalein at pH 12, which is measured at 578 nm. Magnesium and iron are excluded by using 8-hydroxyquinoline. It was found that sample acidity affected the reaction and the buffering capacity had to be increased.

Magnesium at pH 10 gives a colour with calmagite. Calcium is excluded using EGTA. It was found that the calcium interference could only be reduced and not removed, and it was concluded that the method was not suitable when the calcium content exceeds 100 mg/l.

The total hardness method is an adaption of the 'calmagite' method using magnesium disodium EDTA. Calcium releases magnesium from the EDTA salt. Magnesium then reacts with the calmagite at pH 10. The method was found to be linear over the range 80–400 mg/l as $CaCO_3$. Results below this level were done using a low-level method, which was linear from 5–80 mg/l. This was achieved merely by altering the sample size. The method was found to be temperature-dependent and a 37°C temperature bath was used. With no temperature bath the CV was about 5%.

The rest of the chemistries were fairly straightforward. The phosphate method was an adaption of the ammonium molybdate method. The only problem encountered was a tendency for the blue colour formed to coat the colorimeter. The addition of acetone to the ascorbic acid cured this problem.

The sulphate method was turbidimetric, using gelatin to keep the precipitate in suspension.

The chloride method was the standard ferric nitrate and mercuric thiocyanate procedure.

The nitrite method is the same as the nitrate method without the reductant step.

### *Precision*

As can be seen from table 1, the IQAS gives excellent performance characteristics. These are considerably better than for the autoanalyser systems in use in this laboratory. They were also achieved without any special techniques like curve regeneration etc. The CVs for the within-batch standard deviation obtained at the 75% level varied from 0·2 to 0·7, which compared very favourably with the 2–5% obtained with the autoanalyser systems.

The harmonized monitoring tests (table 2) show that the between-batch standard deviation is larger than the within batch and is in some cases significantly so. But, as can be seen from the table, the criterion that the total standard deviation of a single observation should not be greater than 5% of the concentration or 0·025 (whichever is the greater) is easily met, i.e. $St^2/Z^2$ is much less than the calculated values for F 0·05. It can be seen that in this context the between-batch standard deviation is only significant compared to the within-batch standard deviation because the within-batch standard deviation is so small.

Table 4 shows the CVs for the total error (both within- and between-batch standard deviation) on those determinands more regularly analysed. The CV is approximately double the within-batch CV in most cases. These are still extremely good figures.

### *Carry-over*

Carry-over was in most cases practically indetectable (see table 1). Again, this was a considerable improvement on figures obtained on the autoanalyser systems (2–5%). The only method to show carry-over was the phosphate method (0·72%); this is because the reaction mixture is fairly viscous and clings to the mixers.

### *Detection limits*

The detection limits obtained (see table 1) were an order of magnitude better than those obtained on the autoanalyser systems. Recoveries were excellent, showing no matrix effects.

### *Accuracy of analysis*

From table 4 it can be seen that the mean control values are very close to the actual values. Table 3 indicates that the mean percentages differences between the two methods are very small. The tests show no apparent differences between the two sets of results, except for the phosphate method. This method was investigated and the autoanalyser proved to be giving 130% recoveries.

The *t*-test for TON and chloride show some significance for differences between methods, but looked at in context have no practical meaning, i.e. with such a large number of tests of such precision the two sets are different but the difference is only 0·58 and 1·1% respectively and is therefore not significant.

### *Advantages of IQAS over previous techniques*

(1) There is a definite and distinctly identifiable saving in manpower. The speed and ease of use enable one operator to do what two would do using dynamic flow systems.
(2) There is a large saving in space compared to autoanalyser systems. With the current high cost of laboratory space this is a significant factor.
(3) Reagent costs are considerably lower.
(4) The precision, accuracy and bias are an order of magnitude better than those obtained on the autoanalysers presently installed in the authors' laboratory.
(5) There is a large saving in running time as there is no 'start-up time' or 'shut-down time', and, as a consequence of reduced carry-over, the number of repeats is much reduced.
(6) The system is sufficiently versatile to deal with both large sample numbers with many determinands per sample and two or three samples requiring relatively few determinands.

### *Maintenance*

Daily maintenance is simple, involving only the washing down of the mixers and the sample and transfer probes. A simple cleaning program is built into the computer, such that protosol detergent is sampled first, then water, so that the parts in contact with liquid are cleaned. It was found necessary to wash the phosphate lines daily using 'Protosol', as the phosphate reagent was unstable and coated the tube if left overnight.

The water baths are changed weekly and the dispense points are washed. On a rotation basis it was found to be good practice to wash with 'Protosol' those lines connecting reagent bottles to dispensing points.

### *Machine performance*

Downtime was minimal in the six months of running the IQAS. The software adequately covers most situations that arise. One small point is that it is possible to set up index times which are shorter than the time taken to sample without the computer indicating this. This must be taken account of when setting up a method.

### General

The instrument is simple to operate and easy to use. It is very easy to set up chemistries and alter reaction conditions to optimize chemistries. No problems have occurred with reagent or sample line blockages or deterioration of these lines.

Reagent consumption is very low. Various errors are brought to the operator's attention which have proved very useful. The 'P' message indicates an unsteady colour reading and draws attention to 'high' samples or chipped reaction tubes. In this case the transfer seal cannot pressurize the tube. It also sets off an alarm if the printer is off, or if communication with the laboratory computer is faulty. Any machine malfunctions, i.e. pressure, spectrophotometer lamp failure etc., are also flagged.

### *Disadvantages*

The only real disadvantages are that the IQAS cannot cope with on-line phase separation or distillation. Hence there is no possibility of mounting either the present detergent method involving alkaline methylene blue and chloroform or the cyanide and phenol methods which involve a distillation step.

### Conclusion

In the normal working environment the IQAS has lived up to all expectations, as set out in system advantages with respect to time saving, speed, accuracy and ease of use when linked to a laboratory computer system, the IQAS becomes a very accurate and sophisticated instrument for routine laboratory analysis.

### Acknowledgements

The views expressed in this paper are solely those of the authors. The author would like to thank the Director of Scientific Services of the Severn Trent Water Authority, Mr W. F. Lester, for permission to publish this paper. The authors would also like to thank their colleague, Dr John Cope for undertaking the interfacing of the IQAS with the laboratory data system.

### References

1. Methods for the Examination of Waters and Associated Materials, *Oxidised Nitrogen in Waters* (HMSO, London, 1981).
2. Methods for the Examination of Waters and Associated Materials, *Ammonia in Waters* (HMSO, London, 1981).
3. Methods for the Examination of Waters and Associated Materials, *Chloride in Waters, Sewage and Effluents* (HMSO, London, 1981).
4. Methods for the Examination of Waters and Associated Materials, *Phosphorus in Waters, Effluents and Sewage* (HMSO, London, 1980).
5. Santiago, M. A. *et al.*, *Automated Method for $SO_4$* (American Society for Testing Materials, STP 573, 1975), 35.
6. Methods for the Examination of Waters and Associated Materials, *Silicon in Waters and Effluents* (HMSO, London, 1980).
7. Henry, R. J., *Clinical Chemistry: Principles and Techniques* (Harper and Row, 1974), 656.
8. *Standard Methods for Examination of Water and Waste Water* (New York 1971), 178.

### NOTES FOR AUTHORS

*Journal of Automatic Chemistry* covers all aspects of automation and mechanization in analytical, clinical and industrial environments. The *Journal* publishes original research papers; short communications on innovations, techniques and instrumentation, or current research in progress; reports on recent commercial developments; and meeting reports, book reviews and information on forthcoming events. All research papers are refereed.

### Manuscripts

Two copies of articles should be submitted to the Editor. All articles should be typed in double spacing with ample margins, on one side of the paper only. The following items should be sent: (1) a title-page including a brief and informative title, avoiding the word 'new' and its synonyms; a full list of authors with their affiliations and full addresses; (2) an abstract of about 250 words—this should succinctly describe the scope of the contribution and highlight significant findings or innovations; it should be written in a style which can easily be translated into French and German; (3) the main text with sections and subsections numbered; (4) appendices (if any); (5) references; (6) tables, each table on a separate sheet and accompanied by a caption; (7) illustrations (diagrams, drawings and photographs) numbered in a single sequence from 1 upwards and with the author's name on the back of every illustration; captions to illustrations should be typed on a separate sheet. Papers are accepted for publication on condition that they have been submitted only to *Journal of Automatic Chemistry*.

### References

References should be indicated in the text by numbers following the author's name, i.e. Skeggs [6]. In the reference section they should be arranged thus:

to a journal

Manka, D. P., *Journal of Automatic Chemistry*, **3** (1981), 119.

to a book

Malmstadt, H. V., in *Topics in Automatic Chemistry*, Ed. Stockwell, P. B. and Foreman, J. K. (Horwood, Chichester, 1978), p. 68.

### Illustrations

Line diagrams are preferred to photographs. Original copies of diagrams and drawings should be supplied, and should be drawn to be suitable for reduction to the page or column width of the *Journal*, i.e. to 85 mm or 179 mm, with special attention to lettering size. Photographs may be sent as glossy prints or as negatives.

### Proofs and offprints

The principal or corresponding author will be sent galley proofs for checking and will receive 50 offprints free of charge. Additional offprints may be ordered on a form which accompanies the proofs.

*Manuscripts should be sent to the Editor: Dr Peter B. Stockwell, P.S. Analytical Ltd, 2 Eagles Drive, Tatsfield, Westerham, Kent TN16 2PB, UK.*

